# The Results of Hemivertebra Resection by the Posterior Approach in Children with a Mean Follow-Up of Five Years

**DOI:** 10.1155/2017/4213413

**Published:** 2017-10-15

**Authors:** Ramazan Erden Erturer, Bekir Eray Kilinc, Bahadir Gokcen, Sinan Erdogan, Kursat Kara, Cagatay Ozturk

**Affiliations:** ^1^Department of Orthopedics, Istinye University, Istanbul, Turkey; ^2^Orthopedics Clinic, Golhisar State Hospital, Burdur, Turkey

## Abstract

**Aim:**

To evaluate the radiologic and clinical results of patients who underwent deformity correction and stabilization for congenital spinal deformities using pedicle screws after hemivertebra resection.

**Material and Method:**

Nine patients, mean age 9.2, who underwent posterior hemivertebrectomy and transpedicular fixation for congenital spinal deformity and had longer than five years of follow-up were evaluated retrospectively. The hemivertebrae were located in the thoracic region in 4 patients and thoracolumbar transition region in 5 patients. The patients were evaluated radiologically and clinically in the postoperative period.

**Results:**

Mean length of follow-up was 64.2 months. The mean operating time was 292 minutes. The mean blood loss was 236 mL. The average hospitalization time was 7 days. The amount of correction on the coronal planes was measured as 31%. The mean segmental kyphosis angle was 45.7 degrees preoperatively and it was measured 2.7 degrees in the follow-up period. There were no statistically significant differences between the early postoperative period and final follow-up X-rays with respect to coronal and sagittal plane deformities.

**Conclusion:**

The ability to obtain a sufficient and balanced correction in the cases accompanied by long compensator curvatures that have a structural character in hemivertebra may require longer fusion levels.

## 1. Introduction

Vertebral anomalies may develop due to errors in formation or segmentation or a mixture of both. The type of the anomaly often determines the possibility of progression of the deformity. In particular, nonincarcerated and full segmented hemivertebrae may result in deformities that increase with growth. These deformities may be solely scoliotic or include kyphotic components. On the other hand, it is not possible to estimate the natural course for every case [1–3]. The primary goal in the treatment of congenital spinal deformities is to prevent the progression of the deformity and to provide a healthy growth of the spine. Correction of the already present deformity constitutes a part of the treatment plan in especially cases with severe degrees. Failure to intervene in patients who show a tendency towards progression may result in the formation of deformities which require long fusions and possess neurologic risks [2–5].

Numerous methods have been described for the treatment of congenital scoliosis. Among these hemivertebrae, excision is the only method that enables total correction of the deformity by eliminating the pathology and has predictable results [2, 3, 6]. However, it is a highly demanding technique and may cause neurological complications. While hemivertebra resection can be performed via an anterior-posterior combined approach, it is may be also completed with a posterior approach alone. The posterior-only procedure requires one less incision and it decreased the number of anterior approach-related complications [2].

The aim of this study was to present the results of average period of five-year follow-up in patients, diagnosed with congenital scoliosis and kyphoscoliosis and treated with transpedicular instrumentation and fusion methods after hemivertebra resection with the posterior approach.

## 2. Material and Method

Hemivertebra excisions through a single posterior approach, followed by short or long-segment fusion with transpedicular instrumentation, were performed in 9 children. All patients underwent surgery at our institution between May 2009 and September 2012. There were 4 girls and 5 boys. The mean age at time of surgery was 9.2 years (range, 6–13 years). Among these patients 8 had a single progressive hemivertebra and 1 had 2 progressive hemivertebrae. The hemivertebrae were located in the thoracic region (T3–T11) in 4 patients and thoracolumbar transition region (T12-L1) in 5 patients. Seven affected levels were scoliotic and 2 were kyphoscoliotic.

The diagnosis of hemivertebra was established by clinical, radiographic, CT, and MRI assessment. Standing full spine radiographs will allow the doctor to diagnose the type and severity of the congenital vertebral malformations and track the progression of the curvature over time. An MRI may be ordered to determine if there are spinal cord abnormalities.

Thorough physical, neurological, and radiologic examinations were performed for all patients, and additional systemic and organic anomalies were investigated. All patients underwent preoperative MRI, renal ultrasound, and echocardiography. Of the 8 patients, none had intraspinal anomaly and 1 showed associated anomalies in the genitourinary system.

Radiographic evaluations were made on the preoperative, postoperative, and follow-up standing posteroanterior and lateral radiographs. By using these radiographs, the angles of the curves were measured by the Cobb method. The results of measurements performed on the preoperative and follow-up radiographs were compared statistically. Statistical comparisons were performed with the Mann–Whitney* U* test. *P*   values equal to or less than 0.05 were considered statistically significant.

## 3. Surgical Technique

**Figure 1:**
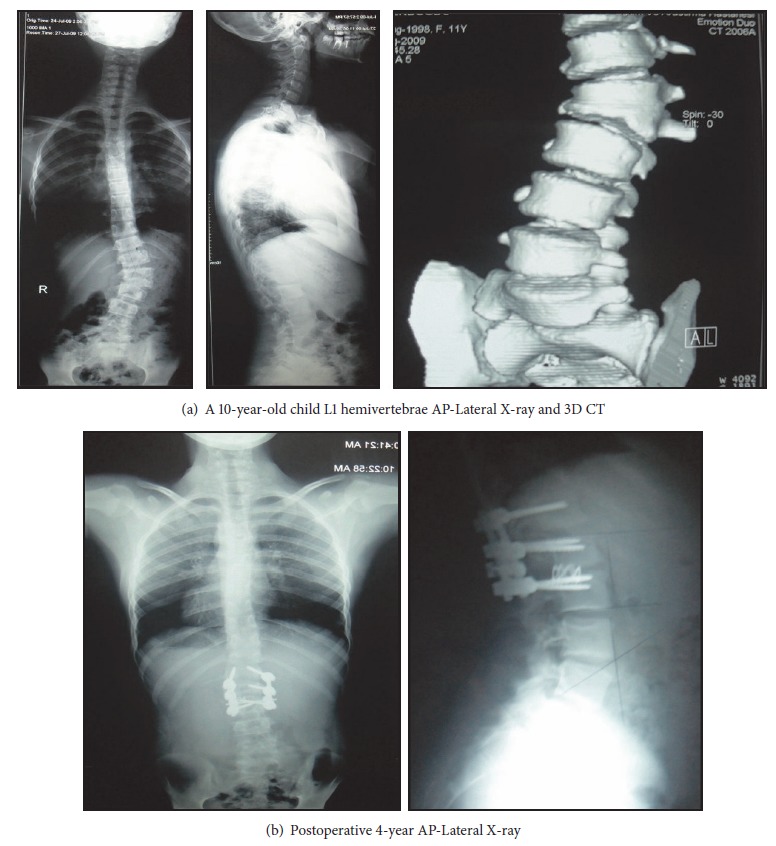


Under general anesthesia, neuromonitoring was performed first; then the hemivertebra field was exposed using a posterior midline incision. One pair of pedicle screws was placed to each of the segments located one level above and below the hemivertebra. The pedicle screws located contralateral to the hemivertebra on the convex part were connected with the temporary rod. The hemivertebra lamina and pedicle were resected with a high speed burr. The spinal cord and the segmental root were protected. The excision was completed by resection of the hemivertebral body. The upper and lower vertebra endplates were abraded and prepared for fusion. An appropriately sized mesh cage was placed on the concave side of the deformity. The deformity was corrected by compression from the convex side aided by the pedicle screws and then checked by X-rays. Following appropriate correction, permanent rods were placed, and the system was locked (Figures 1(a) and 1(b)). For the correction of the deformity, four and more segments were included in the fusion field in three patients The procedure was completed after insertion of grafts to the posterior area. A body cast with femoral extension was applied before termination of anesthesia.

## 4. Results

Mean length of follow-up was 64.2 (range, 54–97) months. The mean operating time was 292 minutes (range, 205–340 minutes). The mean blood loss was 236 mL (range, 180–300 mL). The average hospitalization time was 7 days (range, 5–11 days). Fusion was applied in 3 levels in 4 patients, 2 levels in 2 patients, 4 levels in 1 patient, 10 levels in 1 patient, and 11 levels in one patient. Mean scoliosis angle in scoliotic patients was 40.4 degrees (range, 32–68) before and 12.7 degrees after the operation. Mean segmental kyphosis angle was 45.7 (range, 12–52), which were measured as 2.5 and 2.7 after the operation, respectively (Table 1). There were statistically no significant differences between the early postoperative measurements and final follow-up radiographs with respect to coronal and sagittal plane deformities.

There were no intra- or postoperative neurologic complications, nor any cases of deep or superficial infections. There were no obvious pseudoarthrosis, decompensation, or implant failures at the final follow-up.

## 5. Discussion

Untreated hemivertebra, both full or semisegmented, can lead to serious spinal deformities. It is difficult to predict the natural course of hemivertebrae [1–3]. Generally hemivertebra with contralateral bars are accepted to carry the worst prognosis, followed by hemivertebra at two levels on the same side, single hemivertebra, and wedge vertebra [1].

In case of progressive deformity, nonsurgical methods such as bracing cannot prevent deformity progression. There is an agreement in the literature on the application of surgical treatment in progressive curves [5, 7].

In particular, nonincarcerated hemivertebrae may cause wide and rigid curves in adolescence when left untreated. The location of the hemivertebra is a significant factor in the progression of the deformity. The potential for progression of the curve was found to be higher in pathologies located in the thoracolumbar transition zone or the lumbar area [8]. Prophylactic hemivertebra treatment before the development of compensatory curves or before they possess a structural character is another suggested method. Kyphoscoliotic deformities that reach adulthood become much more difficult to treat problems and are associated with pain, functional insufficiency, and the development of neurologic deficits [8–10]. In our patients, we made the decision for surgery based on the size of the current deformity or its progression.

Various surgical methods have been described for the management of congenital spinal deformities. Among these methods, posterior in situ arthrodesis, anterior and posterior hemiepiphysiodesis, and hemiarthrodesis provide a limited correction. The assessment and estimation of the results are difficult [3]. Although there are reports, which state that hemiepiphysiodesis can be applied successfully, especially in association with instrumented fusion techniques, it is impossible to correct the curves in patients who have developed rigid and structural curves [8, 11]. In a multicentric study, Yaszay et al. compared hemiepiphysiodesis or in situ fusion, instrumented fusion without hemivertebra excision, and instrumented hemivertebra excision. They found that although a higher rate of complications was seen with hemivertebra resection compared to the other two methods, better correction rates were achieved [12]. It is stated that the deformity can be progressively corrected using the recently developed growing rods techniques and may be used in selected patients; however, in a study on this topic, Elsebai et al. reported that complications developed in eight out of the 19 patients [13, 14].

On the other hand, hemivertebra excision is the only method that allows complete correction of the deformity by elimination of the pathology and yields predictable results. Royle first described hemivertebra excision in 1927, and recent studies have proven that it is a reliable and effective method in the treatment of congenital scoliosis [2–5, 15–18]. This method was initially described as the combination of the anterior and posterior approaches performed in one or two stages, yet operations via the posterior approach only have become more predominant recently [2, 3, 19, 20]. In 1991, Ruf and Harms introduced an innovative surgical technique of posterior hemivertebra resection with transpedicular instrumentation, which is especially suitable for early correction in very young children [3]. Either the combined approach or the posterior-only approach provides similar correction rates in the coronal and sagittal planes. In a comparative study, Mladenov et al. obtained similar results with respect to angular measurement, yet the complication rates were lower and the recovery periods were shorter in operations performed by the posterior approach only [4]. Jalanko et al. concluded as a result of their study that PL resection was technically more demanding and slightly faster method for hemivertebral resection. It had nearly as good correction rate as the AP-method but with more minor complications [7]. The posterior-only procedure requires one fewer incision and has decreased the number of anterior approach-related complications [2]. Ruf et al. listed that the advantages of hemivertebra resection by the posterior approach with simultaneous application of transpedicular instrumentation were excellent correction of the curve in the frontal and sagittal planes, early mobilization with high stability, no requirement for anterior access, and low neurologic risk [3, 6].

The average amount of correction in the sagittal and coronal planes we achieved after hemivertebra resection with the posterior-only approach is around 90%. This rate was 54% in the study by Nakamura et al. [20], 75% in the study by Aydogan et al. [19], and 77% in the study by Jalanko et al. [7].

Although there are concerns on the use of pedicle screws in children with growth potential, studies by Ruf et al. showed that the method did not show an adverse effect on the vertebral growth and did not result in any narrowing in the spinal canal. It is suggested that the number of levels included in the fusion site should be kept at a minimum, especially in patients with a high potential for growth. On the other hand, the risk of new deformity development increases in short fusions [3, 6]. Deformities that have developed due to hemivertebrae only and not associated with compensatory curves are amenable to correction by using short segment instrumentation. Therefore management of the deformity by appropriate timing becomes significant. Patients with severe curves in whom compensatory curves have developed require long fusions. In patients who do not have compensator curvature, the deformities could be corrected with short system instrumentation limited to one level above and one below the pathologic segment.

Previous studies have reported that various complications may develop after hemivertebra resection including infection, bleeding, temporary or permanent neurologic injuries, failed instrumentation or recurrence of the deformity [2, 5, 8, 16, 20]. The correction attained during the postoperative period remained stable in all patients, and none of the patients sustained any of the complications reported in the literature.

Previous studies have reported that operations performed by posterior surgery are technically more demanding, and the risk of developing neurologic complications is higher compared to combined surgery [2, 7, 8, 18]. Recent studies have shown significantly lower complications rates [6, 10, 13]. A clear view of the neural structures prior to correction maneuver is recommended to avoid neurologic complications [3]. Neuromonitoring was used routinely in all our cases. Complications such as hemorrhage, bleeding, infections, and recurrence of deformity associated with hemivertebra resection are rarely reported in the literature, but we have not encountered such a negative situation in our series (2-8).

**Figure 2:**
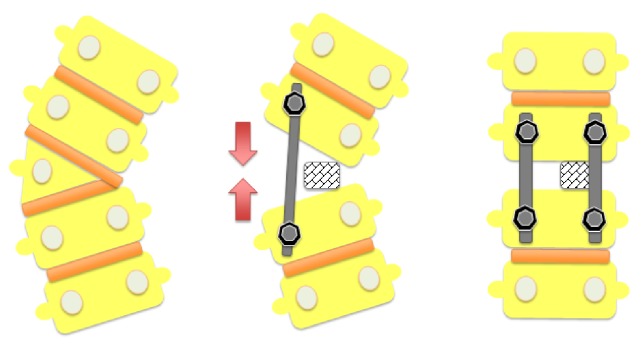
Correction of the coronal plan deformities using mesh cage after hemivertebra resection.

**Figure 3:**
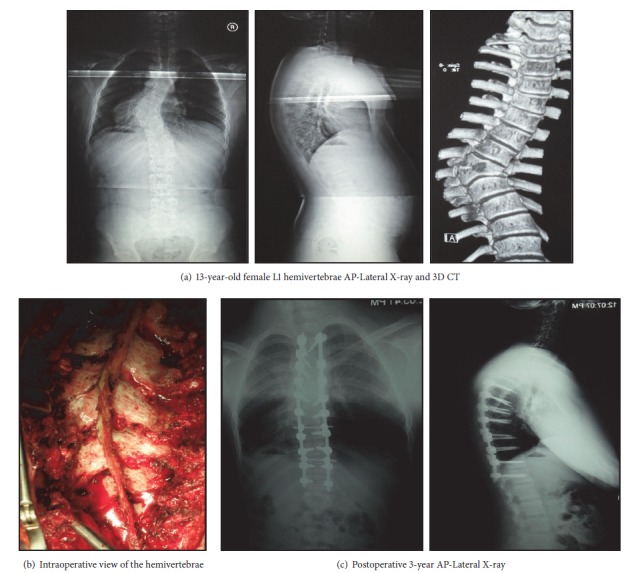


When the fusion level is determined, the size of the curvature and the presence of the accompanying compensatory curvature become important. Correction of deformity should be attempted with a fusion that will take off the least segments (Figure 2). However, in cases of long thoracic or thoracolumbar spines or structural characterization of the compensatory curves, it may be necessary to apply long-segment fusion to correct the deformity (Figures 3(a), 3(b), and 3(c)). Therefore, surgical intervention in progressive cases should be performed at the earliest age.

There were no complications after operations performed under neuromonitoring. In addition to its contribution to fusion, the mesh cage inserted to the level undergoing resection aids in the correction of the deformity by a lever effect. There were no significant recurrences in the coronal and sagittal plane deformities during the follow-up period. These results demonstrate that hemivertebra resection by a posterior approach with segmental posterior transpedicular instrumentation is an effective and safe treatment options in children with congenital spine deformities. Despite the five-year follow-up period, it is appropriate to reevaluate all of the patients at the end of the growth cycle to say that the deformity does not recur.

## Figures and Tables

**Table 1:** Angular values of coronal and sagittal plane deformities preoperative, postoperative, and follow-up period.

	Cobb angle (coronal plane)	Segmental kyphosis angle (sagittal plane)
Preoperative	40.4° (32–68)	45.7° (12–52)
Postoperative	12.7° (5–18)	2.5° (2–6)
Follow-up period	12.5° (5–20)	2.7° (2–6)

## References

[1] McMaster M. J., Singh H. (1999). Natural History of Congenital Kyphosis and Kyphoscoliosis. A Study of One Hundred and Twelve Patients. *The Journal of Bone & Joint Surgery*.

[2] Zhang J., Shengru W., Qiu G., Yu B., Yipeng W., Luk K. D. (2011). The efficacy and complications of posterior hemivertebra resection. *European Spine Journal*.

[3] Ruf M., Harms J. (2002). Hemivertebra resection by a posterior approach: innovative operative technique and first results. *Spine*.

[4] Mladenov K., Kunkel P., Stuecker R. (2012). Hemivertebra resection in children, results after single posterior approach and after combined anterior and posterior approach: a comparative study. *European Spine Journal*.

[5] Holte D. C., Winter R. B., Lonstein J. E., Denis F. (1995). Excision of hemivertebrae and wedge resection in the treatment of congenital scoliosis. *The Journal of Bone & Joint Surgery*.

[6] Ruf M., Jensen R., Letko L., Harms J. (2009). Hemivertebra Resection and Osteotomies in Congenital Spine Deformity. *Spine*.

[7] Jalanko T., Rintala R., Puisto V., Helenius I. (2011). Hemivertebra resection for congenital scoliosis in young children: comparison of clinical, radiographic, and health-related quality of life outcomes between the anteroposterior and posterolateral approaches. *Spine*.

[8] Shono Y., Abumi K., Kaneda K. (2001). One-Stage Posterior Hemivertebra Resection and Correction Using Segmental Posterior Instrumentation. *Spine*.

[9] Winter R. B., Moe J. H., Lonstein J. E. (1984). Posterior spinal arthrodesis for congenital scoliosis. An analysis of the cases of two hundred and ninety patients, five to nineteen years old. *The Journal of Bone & Joint Surgery*.

[10] Wang S., Zhang J., Qiu G., Li S., Yu B., Weng X. (2013). Posterior hemivertebra resection with bisegmental fusion for congenital scoliosis: more than 3 year outcomes and analysis of unanticipated surgeries. *European Spine Journal*.

[11] Alanay A., Dede O., Yazici M. (2012). Convex Instrumented Hemiepiphysiodesis with Concave Distraction: A Preliminary Report. *Clinical Orthopaedics and Related Research®*.

[12] Yaszay B., O'Brien M., Shufflebarger H. L. (2011). Efficacy of hemivertebra resection for congenital scoliosis: a multicenter retrospective comparison of three surgical techniques. *Spine*.

[13] Elsebaie H. B., Kaptan W., El Miligui Y. (2010). Anterior Instrumentation and Correction of Congenital Spinal Deformities Under Age of Four Without Hemivertebrectomy: a New Alternative. *Spine*.

[14] Elsebai H. B., Yazici M., Thompson G. H. (2011). Safety and efficacy of growing rod technique for pediatric congenital spinal deformities. *Journal of Pediatric Orthopaedics*.

[15] Royle N. D. (1928). The operative removal of an accessory vertebra. *Med J Aust*.

[16] Deviren V., Berven S., Smith J. A., Emami A., Hu S. S., Bradford D. S. (2001). Excision of hemivertebrae in the management of congenital scoliosis involving the thoracic and thoracolumbar spine. *The Journal of Bone and Joint Surgery*.

[17] Callahan B. C., Georgopoulos G., Eilert R. E. (1997). Hemivertebral Excision for Congenital Scoliosis. *Journal of Pediatric Orthopaedics*.

[18] Xu W., Yang S., Wu X., Claus C. (2010). Hemivertebra excision with short-segment spinal fusion through combined anterior and posterior approaches for congenital spinal deformities in children. *Journal of Pediatric Orthopaedics B*.

[19] Aydogan M., Ozturk C., Tezer M., Mirzanli C., Karatoprak O., Hamzaoglu A. (2008). Posterior vertebrectomy in kyphosis, scoliosis and kyphoscoliosis due to hemivertebra. *Journal of Pediatric Orthopaedics B*.

[20] Nakamura H., Matsuda H., Konishi S., Yamano Y. (2002). Single-stage excision of hemivertebrae via the posterior approach alone for congenital spine deformity: follow-up period longer than ten years. *Spine*.

